# How to See with the Microscope

**Published:** 1881-06

**Authors:** 


How to see with the microscope. By J. Edwards Smith, M.D. pp. 300 Chicago, Duncan Brothers, 1881.

This excellent manual is designed as a guide for the student, just beginning microscopic practice. It forms a valuable introduction to the more elaborate works of Carpenter and Beale. Written in a "gossipy and common-place" style it gets thoroughly in sympathy with the reader and thus succeeds in giving him a clear understanding of the subject. It is divided into chapters which treat respectively of the stand, the objective, the eye-piece; of illumination and illuminators. Then comes advice to the student about the selection of an instrument. Why there should be included directions for volumetric analysis of urine is not so clear as the microscope has to deal only with deposits. The whole concludes with an appendix and a supplement of odds and ends of much practical value.

The merits of angular aperture are difficult of comprehension for the beginner and indeed for the advanced student also. But the definitions and the discussion of the subject in this book are the clearest we have ever seen. The author is decisively in favor of "high" or "wide" angles. His large experience entitles his opinions to great weight. Indeed even if a student have knowledge enough to be theoretically in favor of the low angles yet when seeking a counselor for more advanced steps it is safer to adopt the vigorous opinions of this author until he shall have acquired practical mastery of the details, when he will be competent to judge for himself. The author, however, neglects to clearly point out the nonsense of talking about an angular aperture of 180 degrees. There is no such thing. Collar adjustment receives the attention its importance deserves. His objections to the binocular are forcible. Yet when an operator is working the polariscope there is some advantage in being able to slip the Wenham prism into place, look down the second tube and judge of the focusing without disturbing the analyzer on the eye-piece. The same may be said of the micro-spectroscope. There are many objections to the placing of fine adjustment upon the nose of the instrument. The eye being engaged, the hand resting on the coarse adjustment has considerable wandering to do before it finds the fine adjustment. It makes it more difficult to steady the hand when working, especially with large instruments. The principle is wrong because it changes the distance between eye-piece and objective. The nose-piece carrying two or more objectives, which is so great a convenience that its use must constantly increase, makes too great a weight for it and causes it to become immovable.

There is a very good notice of the comparatively new accessories—the Woodward Illuminator and the Tolles' Traverse Lens. The letters, N. and L. in the wood cut of the latter are very obscure; they should be removed to a more conspicuous position.

On page 211, the sentence "every one of whom have expressed their satisfaction," contains two violations of grammar. Let it be corrected in the next edition. There is also a word left out in the ninth line, page 221. These, however, are small errors and in no way detract from the value of the book. To every one who thinks of studying the microscope practically, we say let him not spend a single dollar for an instrument until he shall have thoroughly read this little volume.

